# Risk factors for primary Sjögren’s Syndrome: a systematic review and meta-analysis

**DOI:** 10.1007/s10067-022-06474-8

**Published:** 2022-12-19

**Authors:** Liang Jin, Min Dai, Chengyin Li, Jing Wang, Bin Wu

**Affiliations:** 1 Department of Rheumatology, Chongqing Hospital of Traditional Chinese Medicine, No. 6, Pan Xi Qi Zhi Road, Jiangbei District, Chongqing, 400021 China; 2 Shenzhen Hospital of Guangzhou University of Traditional Chinese Medicine, No.6001, Beihuan Avenue, Futian District, Shenzhen, 518000 China

**Keywords:** Meta-analysis, Primary Sjögren’s syndrome, Risk factor, Systematic review

## Abstract

**Objectives:**

The aim of this study was to analyze the risk factors for primary Sjögren’s Syndrome (pSS) by conducting a meta-analysis of observational studies.

**Methods:**

Four electronic databases were searched from inception to August 2022. The search strategy included medical subject headings (MeSH) and text words. Outcomes were calculated and reported as the odds ratio (OR) and 95% confidence interval (CI).

**Results:**

Twelve studies consisting of nine case–control and three cohort studies were analyzed. Significant positive relationships between infection, a family history of autoimmune disease in first-degree relatives, negative stressful life events, CGGGG insertion/deletion polymorphisms in the IRF5 gene and the onset of pSS were found, with pooled ORs and 95% CIs of 2.73 (1.93, 3.86), 5.93 (3.34, 10.52), 1.69 (1.27, 2.24) and 2.69 (1.97, 3.66), respectively. In contrast, the results showed that a history of smoking was not associated with the onset of pSS, with a pooled OR and 95% CI of 1.39 (0.76, 2.53). However, a statistically significant negative association between current smoking and pSS was detected, with a pooled OR and 95% CI of 0.4 (0.29, 0.83).

**Conclusions:**

Our research indicated that infection, a family history of autoimmune disease in first-degree relatives, negative stressful life events and CGGGG insertion/deletion polymorphisms in the IRF5 gene might be risk factors for pSS. In contrast, our study demonstrated that a history of smoking was not associated with the onset of pSS, whereas current smoking was negatively associated with pSS onset.

**Systematic review registration:**

We registered this review on INPLASY (https://inplasy.com/ ) under registration number INPLASY202230005.

**Supplementary Information:**

The online version contains supplementary material available at 10.1007/s10067-022-06474-8.

## Introduction

Primary Sjögren’s syndrome (pSS) is a chronic and heterogeneous disorder characterized by a wide spectrum of glandular and extra-glandular features, leading to mononuclear cell infiltration of exocrine glands, notably the lacrimal and salivary glands [1–3]. It is a systemic autoimmune disease (AD) with prevalence ranging from 0.03 to 5% in different countries, which is the second highest AD in China [4, 5]. Most patients with pSS suffer from dry eyes, dry mouth and fatigue, which inevitably leads to poor quality of life. In at least one-third of patients, there may be associated extraglandular manifestations, such as renal tubular acidosis and interstitial lung disease, which could exacerbate the symptoms and even have a lethal impact.

Although pSS is a complex disease, with genetic and epigenetic factors potentially contributing to its occurrence, there is still no consensus among experts regarding the risk factors and pathogenesis of pSS. An increasing number of studies have been performed in recent years to improve our understanding of pSS, and potential risk factors have been identified, which include various types of infection, genetic susceptibilities and some environmental factors [6, 7]. One study reviewed the genetic analyses published to date to confirm our current understanding of the epigenetic mechanisms involved in pSS [8]. Utomo and Putri identified and analyzed the role of infection in the development of pSS based on various published research articles [9]. Several other studies have suggested that potential risk factors, such as hepatitis C virus (HCV), miRNA-146a, tumor necrosis factor and cigarette smoking, might be associated with pSS [10–12].

To date, a meta-analysis that comprehensively summarizes the risk factors of pSS based on existing evidence has been lacking. Here, we analyze the risk factors in patients with pSS by conducting a meta-analysis of observational studies.

## Materials and methods

### Search strategy

A systematic review of the electronic databases (PubMed, Embase, Cochrane library, Web of Science) was conducted independently by two authors from their inception to August 30, 2022, without any language restrictions. We also searched ClinicalTrials.gov^1^ for unpublished reports. Further studies were searched in the reference lists of the studies identified. We also contacted the author when necessary. To ensure a comprehensive search, the search strategy included medical subject headings (MeSH) terms and text words: “Sjögren’s Syndrome” (Major), “risk factors” (MeSH) and “case–control study” (MeSH). Boolean logic operators, position operators and truncation symbols were used to combine search terms. Any discrepancies were resolved by consensus.

### Study selection

The inclusion criteria for considering studies for this review were as follows: (a) the design is a case–control study, cohort study or cross-sectional study based on unrelated individuals; (b) study published in full-text form; (c) patients meet the diagnostic criteria according to the American–European classification criteria [13]; (d) study focuses on the risk factors for pSS; (e) the application of statistical methods is specific and appropriate (i.e. logistic regression analysis) and (f) specific data are provided, including an odds ratio (OR) with its 95% confidence interval (CI), or sufficient data are available to calculate the OR and its CI. Studies were excluded if one of the following statements applied: (a) sample size < 50; (b) patients did not meet the American–European classification criteria; (c) inappropriate statistical methods and (d) insufficient overlapping data. Any disagreement was resolved through discussion or, if required, a third assessor was consulted.

### Risk of bias assessment

The risk of bias among the included studies was assessed according to the Newcastle–Ottawa quality assessment scale (NOS) [14]. A study can be awarded a maximum of nine stars according to items within the Selection, Exposure and Comparability categories. A study awarded six or more stars was recognized as a high-quality study. Sensitivity analysis was also conducted to assess the impact of the risk of bias.

### Data extraction and management

A standardized form was designed for this meta-analysis. Two authors independently extracted data using the form to identify eligible studies. The following information was collected from each study: authors, year of publication, study design, statistical method, risk factors (including the hazard ratio or OR and 95% CI of each risk factor) and number of cases and controls. Discrepancies were resolved by discussion and by consultation with other authors. When the necessary information was unclear, we attempted to contact the authors of the original reports to provide further details.

### Statistical analysis

The synthesis, calculation and analysis of extracted data were all performed using Review Manager 5.3 and STATA14 software. Pooled statistics were calculated as ORs with 95% CIs. Assessment of statistical heterogeneity was conducted using Cochran’s Q statistic, and Higgins and Thompsons’ *I*^2^. The fixed-effects model was used for meta-。analysis when *p* ≥ 0.10 and *I*^2^ ≤ 50%, which indicated that the homogeneity was appropriate. Otherwise, the random-effects model was used. Funnel plots, Egger’s regression asymmetry tests and sensitivity analysis were used to analyze potential publication bias and to test the stability of the results of the meta-analysis. If required, for each risk factor, the final-effect ORs and 95% CIs were pooled by means of both random and fixed-effect models, and the results were compared. When the number of included studies was greater than five, studies with a significant deviation from the 95% CI in the funnel plot were excluded from the meta-analysis, and the results were compared with those contained in all included studies. *p* values < 0.05 were considered statistically significant.

## Results

### Results of literature search

**Fig. 1:**
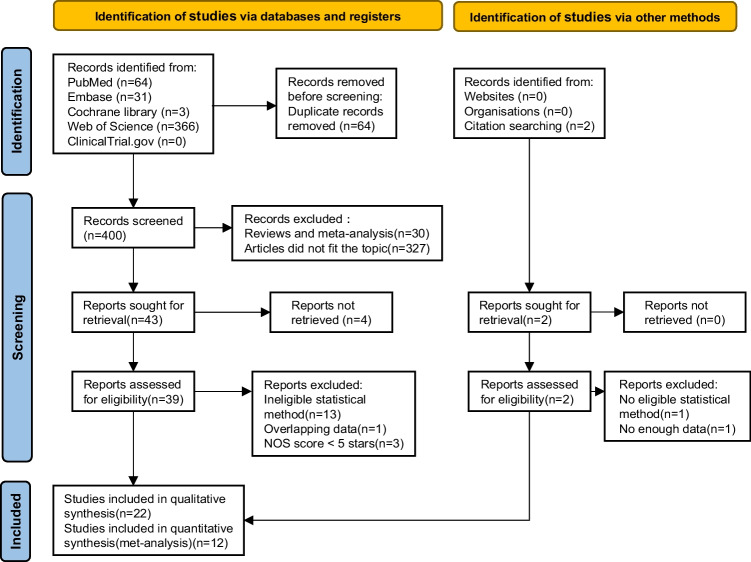
Flow chart of screening articles for inclusion in the study

A total of 464 relevant citations were retrieved through electronic databases and other search sources (PubMed 64, EMBASE 31, Cochrane library 3, Web of Science 366 and other search sources 0), of which, 64 citations were duplicates. After excluding all ineligible articles and evaluating 41 report, 22 studies [15–36] were included in qualitative synthesis, of which, 12 studies [25–36] were included in the meta-analysis. All these studies were published in English and the details of screening, and the number of records identified, included and excluded is illustrated in the study flow diagram (Fig. 1). A complete list of the 19 reports that were excluded due to methodological and other limitations is given in Supplementary table 1 (p 1–2).

### Characteristics and quality assessment of the included studies

The 12 studies included nine case–control studies and three cohort studies. A total of 158,539 research objects were recruited, including 18,215 cases and 140,324 healthy controls. Control groups in 11 studies were sex-matched. All studies were scored using the NOS, and the results showed that four studies [25, 31, 32, 35] scored eight stars (high quality), three studies [26, 28, 29] scored seven stars and five studies [27, 30, 33, 34, 36] scored six stars, indicating that the overall quality of the included articles was eligible. The methodological quality assessment of all included studies is illustrated in Supplementary table 2 (p 3). Potential risk factors were identified based on the consistency of risk factors studied in the included literature. Finally, six potential risk factors, namely infection, a history of smoking, a family history of AD in first-degree relatives (FDR), a history of pregnancy, CGGGG insertion/deletion (Indel) polymorphisms in the IRF5 gene and the number of negative stressful life events > 1, were included in the meta-analysis. The main characteristics of the 12 studies are shown in Table 1. The main characteristics of the 10 studies [15–24] included in qualitative synthesis only are shown in Table 2.

**Table 1:** Information on the studies included in the meta-analysis

Author, year of publication	Country	Study design	Diagnose criteria	No. of cases	No. of control	Sex comparability(Y/N)	Potential risk factor	NOS score (stars)
FanYan Meng et al. 2021 [25]	China	Case–control study	AECG criteria	67	67	Y	5,7	8
Johannes mofors et al. 2020 [26]	Sweden	Case–control study	AECG criteria	815	4425	Y	2,3	7
Hadas Ben-Eli et al. 2019 [27]	Jerusalem	Case–control study	AECG criteria	91	211	Y	1,3,4	6
Luisa Servioli et al. 2019 [28]	USA	Cohort study	AECG criteria	106	318	Y	2	7
J.mofors et al. 2019 [29]	Sweden	Case–control study	AECG criteria	945	9048	Y	1	7
Peter Olsson et al. 2017 [30]	Sweden	Case–control study	AECG criteria	63	252	Y	2,3	6
Wen-Cheng Chao et al. 2017 [31]	China	Case–control study	AECG criteria	5741	86,265	Y	1	8
Chih-Ching Yeh et al. 2016 [32]	China	Case–control study	AECG criteria	9629	38,516	Y	1	8
Corinne MR et al. 2009 [33]	French	Cohort study	AECG criteria	200	282	N	6	6
G Nordmark et al. 2009 [34]	Sweden, Norway	Cohort study	AECG criteria	368	711	N	6	6
D Karaiskos et al. 2009 [35]	Athens	Case–control study	AECG criteria	47	120	Y	7	8
R Priori et al. 2007 [36]	Italy	Case–control study	AECG criteria	143	109	Y	2,3,4,5	6

1, infection; 2, a history of smoking; 3, current smoking; 4, a family history of AD in FDR; 5, a history of pregnancy; 6, the CGGGG Indel polymorphism in the IRF5 gene; 7, the number of negative stressful life events > 1

Y: yes; N, no or not mentioned

AECG criteria: criteria proposed by the American–European Consensus Group

**Table 2:** Information on the studies included for qualitative synthesis only

Author, year of publication	Country	Study design	Diagnostic criteria	No. of cases	No. of control	Sex comparability (Y/N)	Potential risk factor	Correlation with pSS	NOS score (stars)
A. Machowicz et al. 2020 [15]	UK	Cohort study	AECG criteria	82	21	Y	Mediterranean diet	Inverse	7
McCoy Sara S et al. 2020 [16]	USA	Case–control study	AECG criteria	1320	1360	Y	Reduced lifetime sex hormone exposure	Significant	6
Wen-ChengChao et al. 2018 [17]	Taiwan	Case–control study	AECG criteria	5553	83,295	Y	Antibiotics for NTM infection	Significant	8
Ming-Chi Lu et al. 2016 [18]	Taiwan	Case–control study	AECG criteria	360	1800	Y	Irregular menstrual cycles	Significant	8
Yan Du et al. 2015 [19]	China	Case–control study	AECG criteria	403	2169	Y	Functional LILRA3	Significant	6
Mengru Liu et al. 2015 [20]	China	Case–control study	AECG criteria	476	1278	N	DCIR SNP rs2377422	Significant	5
Fei Sun et al. 2013 [21]	China	Case–control study	AECG criteria	555	597	Y	TNFSF4, TNFAIP3 and FAM167A-BLK	Do not exist or are very weak	7
JOHANNES C. NOSSENT et al. 2012 [22]	Australia	Cross-sectional study	AECG criteria	174	162	N	FCGR3B CN	Significant	5
N Gestermann et al. 2010 [23]	France	Cross-sectional study	AECG criteria	368	711	N	STAT4 rs7582694 C allele	Significant	5
Behrouz Mostafavi et al. 2005 [24]	Malmö	Case–control study	AECG criteria	47	120	Y	High birth weight and low maternal age	Significant	8

Y, yes; N, no or not mentioned

*AECG* American and European Consensus Group

### Meta-analysis of potential risk factors

**Fig. 2:**
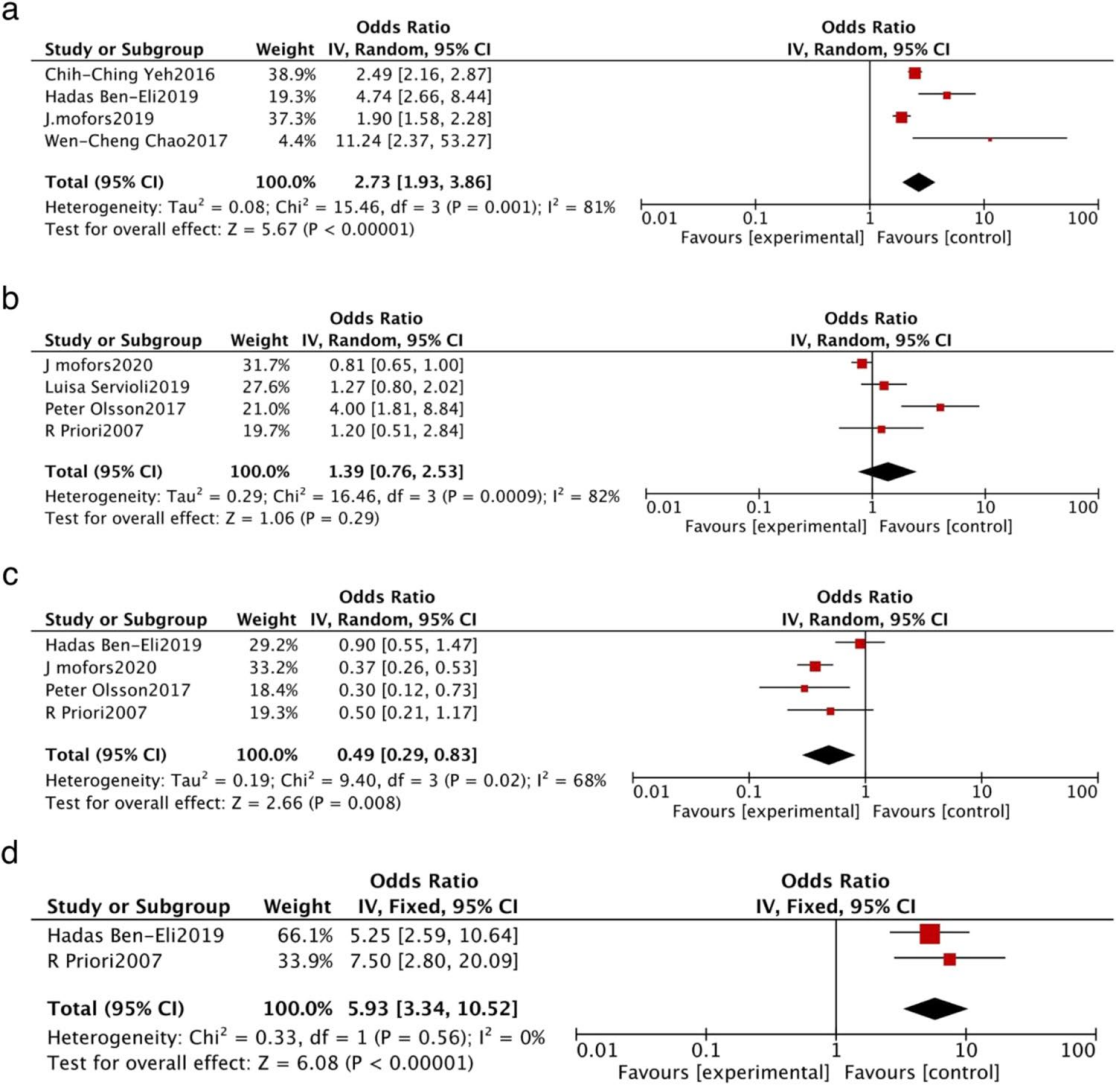
Forest plot of studies based on certain variables. The variables were as follows: (**a**) current infection, (**b**) a history of smoking, (**c**) currently smoking, (**d**) a family history of autoimmune disease (AD) in first-degree relatives (FDRs)

**Fig. 3:**
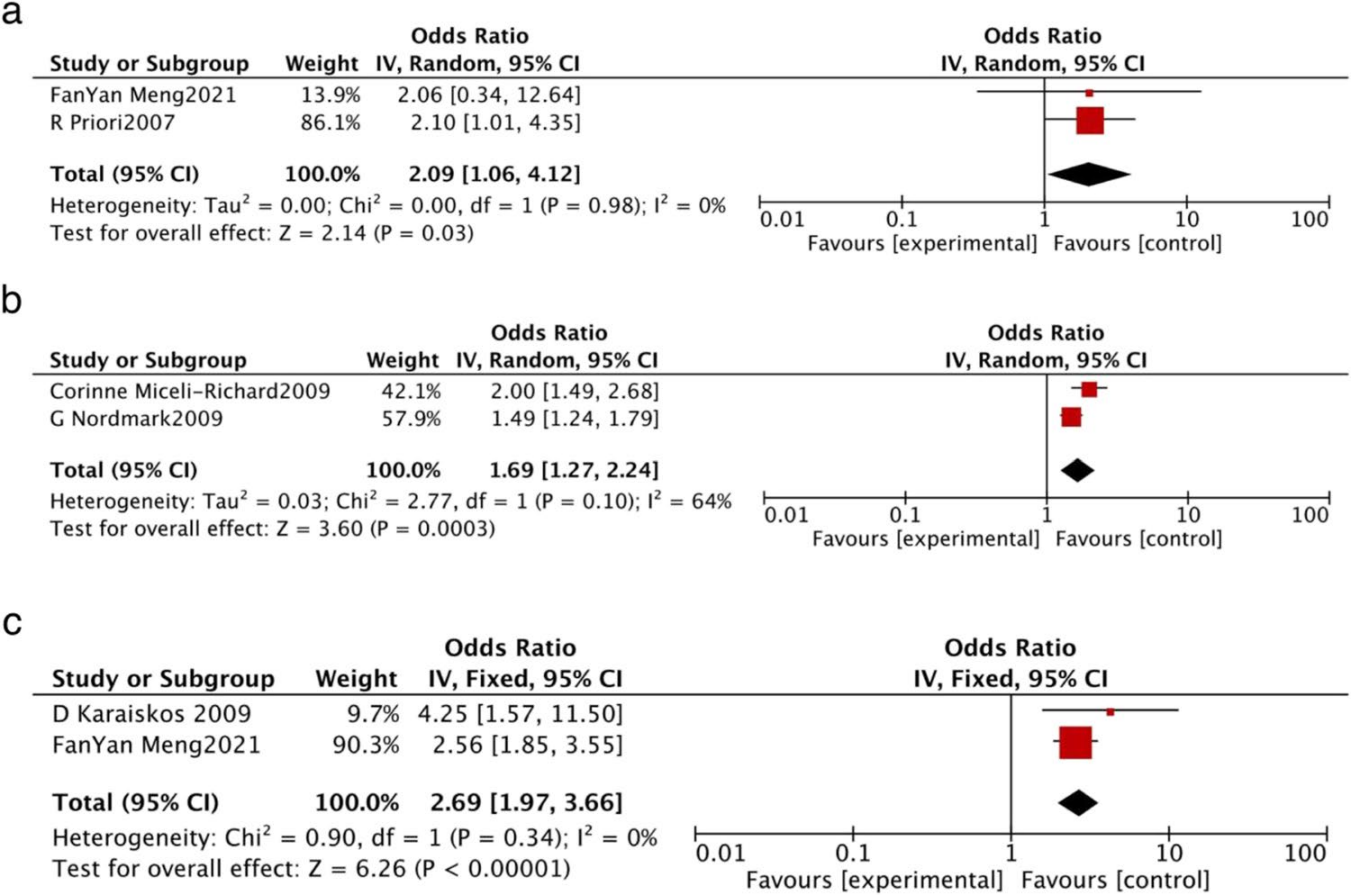
Forest plot of studies based on certain variables. The variables were as follows: (**a**) a history of pregnancy, (**b**) CGGGG insertion/deletion (Indel) polymorphisms in the IRF5 gene and (**c**) negative stressful events

The 12 included studies examined associations between several factors and pSS. The original data regarding the six selected factors (infection, a history of smoking, a family history of AD in FDR, a history of pregnancy, CGGGG indel polymorphisms in the IRF5 gene and the number of negative stressful life events) were pooled using the random-effects model or fixed-effects model based on the results of the heterogeneity test. The details of these analyses are provided in Table 3, Figs. 2 and 3.

**Table 3:** Original data and the results of meta-analysis

Potential risk factor	No. of included studies	Included studies	OR/RR (95% CI)	Heterogeneity		Model	Results of meta-analysis		
				*I*^2^ (%)	*p* value		Pooled OR (95%CI)	*p* value	*Z*
Infection	4	Hadas Ben-Eli2019 [27]	4.74 (2.66, 8.44)	81	0.001	Random	2.73 (1.93, 3.86)	< 0.00001^**^	5.67
		J. mofors2019 [29]	1.9 (1.6, 2.3)						
		Wen-Cheng Chao2017 [31]	11.24 (2.37,53.24)						
		Chih-Ching Yeh2016 [32]	2.49 (2.16,2.86)						
Former smoking	4	J mofors2020 [26]	0.81 (0.65, 1)	82	0.0009	Random	1.39 (0.76, 2.53)	0.29^△^	1.06
		Luisa Servioli2019 [28]	1.27 (0.8, 2.03)						
		Peter Olsson2017 [30]	4 (1.8, 8.8)						
		R Priori2007 [36]	1.2 (0.5, 2.8)						
Current smoking	4	Hadas Ben-Eli2019 [27]	0.37 (0.26,0.53)	68	0.02	Random	0.49 (0.29,0.83)	0.008^**^	2.66
		J. mofors2019 [29]	0.9 (0.55,1.47)						
		Peter Olsson2017 [30]	0.30 (0.1,0.6)						
		R Priori2007 [36]	0.5 (0.2,1.1)						
A family history of AD in FDR	2	Hadas Ben-Eli 2019 [27]	5.25 (2.59, 10.63)	0	0.56	Fixed	5.93 (3.34, 10.52)	< 0.00001^**^	6.08
		R Priori 2017 [36]	7.5 (2.8, 20.1)						
Pregnancy history	2	FanYan Meng2021 [25]	2.06 (0.34,12.64)	0	0.98	Fixed	2.09 (1.06,4.12)	0.03^*^	2.14
		R Priori2007 [36]	2.10 (1.01,4.35)						
The CGGGG Indel polymorphisms in IRF5	2	Corinne Miceli-Richard 2009 [33]	2 (1.5, 2.7)	64	0.10	Random	1.69 (1.27, 2.24)	0.0003^**^	3.60
		G Nordmark2009 [34]	1.49 (1.24, 1.79)						
The number of negative stressful life events > 1	2	FanYan Meng2021 [25]	2.56 (1.85, 3.55)	0	0.34	Fixed	2.69 (1.97, 3.66)	< 0.00001^**^	6.26
		D Karaiskos 2009 [35]	4.25 (1.57, 11.49)						

^*^p < 0.05, **p < 0.01, ^△^*p* > 0.05

Four studies [28, 29, 31, 32] reported an association between infection and pSS, with significant heterogeneity (*I*^2^ = 81%, *p* = 0.0001). Using a random effects model, we found a statistically significant positive association between infection and pSS, with a pooled OR and 95% CI of 2.73 (1.93, 3.86) (*z* = 5.67, *p* < 0.00001, Fig. 2a).

Four studies [26, 28, 30, 36] examined the association between a history of smoking and pSS. There was significant heterogeneity (*I*^2^ = 82%, *p* = 0.0009) so a random-effects model was used, and the results revealed no statistical association between a history of smoking and pSS, with a pooled OR and 95% CI of 1.39 (0.76, 2.53) (*z* = 1.06, *p* = 0.29, Fig. 2b).

Four studies [27, 28, 30, 36] examined the association between current smoking and pSS. There was significant heterogeneity (*I*^2^ = 68%, *p* = 0.02) so a random effects model was used, and the results revealed a statistically significant negative association between current smoking and pSS, with a pooled OR and 95% CI of 0.49 (0.29, 0.83) (*z* = 2.66, *p* = 0.008, Fig. 2c).

Two studies [28, 36] examined the association between a family history of AD in FDR and pSS. There was no heterogeneity (*I*^2^ = 0, *p* = 0.56) so a fixed-effects model was used, and the results revealed a statistically significant positive association between a family history of AD in FDR and pSS, with a pooled OR and 95% CI of 5.93 (3.34, 10.52) (*z* = 6.08, *p* < 0.00001, Fig. 2d).

Two studies [25, 36] examined the association between a history of pregnancy and pSS. There was no heterogeneity (*I*^2^ = 0, *p* = 0.98) so a fixed-effects model was used, and the results revealed a statistically significant positive association between a history of pregnancy and pSS, with a pooled OR and 95% CI of 2.09 (1.06, 4.12) (*z* = 2.14, *p* = 0.03, Fig. 3a).

Two studies [33, 34] examined the association between CGGGG Indel polymorphisms in the IRF5 gene and pSS. There was significant heterogeneity (*I*^2^ = 64%, *p* = 0.1) so a random effects model was used, and the results revealed a marginally significant association between CGGGG Indel polymorphisms in the IRF5 gene and pSS, with a pooled OR and 95% CI of 1.69 (1.27, 2.24) (*z* = 3.60, *p* = 0.0003, Fig. 3b).

Two studies [25, 35] reported the association between the number of negative stressful life events being > 1 and pSS. There was no heterogeneity (*I*^2^ = 0, *p* = 0.34) so a fixed-effects model was used, and the results revealed a statistically significant positive association between the number of negative stressful life events being > 1 and pSS, with a pooled OR and 95% CI of 2.69 (1.97, 3.66) (*z* = 6.26, *p* < 0.00001, Fig. 3c).

### Sensitivity analysis and publication bias

We used STATA 14 software to perform sensitivity analysis. We also compared the results calculated by the fixed-effects model and the random effects model. No obvious significant difference was detected between the results generated using these methods, which indicated that the results of our meta-analysis were stable. The specific data are shown in Table 4.

**Table 4:** Comparison of the results from the fixed-effects model and the random effects model

Potential risk factor	Fixed-effected model		Random-effected model	
	Pooled OR(95%CI)	*p* value	Pooled OR(95%CI)	*p* value
Infection	2.33 (2.09,2.60)	< 0.00001	2.73 (1.93, 3.86)	< 0.00001
Former smoking	0.97 (0.80,1.16)	0.72	1.39 (0.76, 2.53)	0.29
Current smoking	0.48 (0.37,0.62)	< 0.00001	0.49 (0.29,0.83)	0.008
A family history of AD in FDR	5.93 (3.34, 10.52)	< 0.00001	5.93 (3.34, 10.52)	< 0.00001
Pregnancy history	2.09 (1.06,4.12)	0.03	2.09 (1.06,4.12)	0.03
The CGGGG Indel polymorphisms in IRF5	1.62 (1.38,1.89)	< 0.00001	1.69 (1.27, 2.24)	0.0003
The number of negative stressful life events > 1	2.69 (1.97, 3.66)	< 0.00001	2.69 (1.97, 3.66)	< 0.00001

No visual inspection of funnel plots was performed for publication bias indicators because fewer than 10 studies were analyzed. Hence, publication bias in the studies that reported associations between infection, a history of smoking and current smoking with pSS was examined by applying Egger’s regression tests. The results indicated a low possibility of publication bias in this meta-analysis, with *p* values of 0.310, 0.161 and 0.946, respectively.

## Discussion

To the best of our knowledge, this meta-analysis is the first to summarize the potential risk factors of pSS based on related case–control studies and cohort studies. Therefore, both the core findings and the limitations of this research deserve further exploration.

### Infection

Our results demonstrated that infection was a risk factor for pSS. This finding was consistent with previous research examining the association between various infections and the risk of pSS. This link has repeatedly been reported, which suggests that both viruses and bacteria could trigger the onset of pSS [37]. The mechanism involved may be that infection induces inflammation, leading to functional impairment of the affected organs and an over-stimulated immune system. In recent years, several studies have examined the association between different viruses and bacteria and pSS. Viruses have commonly been considered one of the major exogenous factors implicated in the etiopathogenesis of Sjögren’s Syndrome (SS), with HCV being proposed as the principal causative agent in one study [32] included in our meta-analysis. Dinescu et al. reported the cases of two female patients diagnosed with HCV chronic infection, who were later diagnosed with HCV-induced SS [38]. They found that in patients with HCV-induced SS, the core pathophysiological phenomenon was viral-induced sialadenitis. Brito-Zerón et al. conducted a study to analyze 783 Spanish patients with SS and found HCV infection in 13% of these patients [39]. Fewer studies have been conducted regarding bacterial infection and pSS. A study [31] included in our meta-analysis revealed a significant association between a history of non-tuberculous mycobacterial infection and pSS, and a meta-analysis by Chen et al. suggested a significantly higher *Helicobacter pylori* infection rate among patients with SS [40].

### A history of smoking and current smoking

Based on previous studies, smoking, which is a well-established risk factor in ADs, such as rheumatoid arthritis and multiple sclerosis [41, 42], has not been thoroughly studied in pSS. Existing reports present divergent data. Therefore, our study analyzed and summarized the eligible literature on the association between smoking and pSS in detail. Most literature subdivided the research subjects into former smokers and current smokers. On the one hand, in the four studies included in our meta-analysis, the consensus was that current smoking was negatively associated with pSS, with all ORs being < 1 [26, 27, 30, 36]. The pooled results demonstrated that the correlation between current smoking and pSS was negative, which indicated that current smoking might be a protective factor for pSS. On the other hand, the results regarding former smoking were discrepant. Olsson et al. and Mofors et al. found that former smoking was associated with a higher risk of later developing pSS [26, 30], whereas the other two studies indicated the opposite [28, 36]. Therefore, all four studies were included in this meta-analysis, the results of which indicated that former smoking was not associated with the onset of pSS.

It has been established that some immune-mediated chronic inflammatory diseases, such as ulcerative colitis and Behcet’s disease, are less frequent among smokers and may flare up after a patient has quit smoking [43]. While several studies demonstrated that smoking might be protective against pSS [30, 44], our results found that both former smoking and current smoking had no obvious bearing on pSS. Notably, Olsson et al. found that individuals who later developed pSS smoked the same amount in early life as the general population but were more likely to quit smoking [26]. Therefore, the differences in the links between former smoking and current smoking and pSS may in fact reflect early pathological changes, highlighting the chronic, insidious but progressive nature of pSS. In conclusion, the possibility of a protective effect of cigarette smoking on pSS should not be emphasized, with earlier diagnosis and treatment being far more important.

### A family history of AD in FDR and a history of pregnancy

Our results showed that a family history of AD in FDR had a close correlation with the onset of pSS. R Priori et al. found that autoimmune thyroid disease was the most prevalent AD among the FDR of SS patients followed by undifferentiated connective tissue disease, rheumatoid arthritis, systemic lupus erythematosus, insulin-dependent diabetes, psoriasis, seronegative polyarthritis and other ADs [36]. Hadas Ben-Eli et al. did not explicitly elaborate on AD in FDR of SS patients in the study [27]. To our knowledge, there are only a limited number of epidemiologic studies investigating the association between the onset of pSS and the prevalence of AD in the FDR of SS patients. In addition to the two studies included in our meta-analysis, a cohort study in Taiwan also reported that individuals with a family history of AD in FDR were at increased risk of SS [45]. Although we did not include the data in this study because of the ineligible statistical methods used, this result might also be a valuable reference. Some other reports have previously described familial aggregation of ADs in SS patients [46].

Our results demonstrated that a history of pregnancy is weakly correlated with the onset of pSS, suggesting more evidence is needed. This might be attributed to changes in levels of estrogen and progesterone in patients, while further research is needed to study whether a history of pregnancy is directly related to the development of pSS. Mostafavi et al. found that high birth weight and younger maternal age were linked with an increased risk of developing SS in mid-life [24]. Possible mechanisms include modulation of the immune system early in life. And they also pointed that birth weight may be a marker for qualitative and/or quantitative differences in the immune system.

### The CGGGG indel polymorphism in the IRF5 gene

The association between the CGGGG indel polymorphism in the IRF5 gene and pSS was confirmed in our study. The IRF5 gene is implicated in interferon (IFN) secretion after stimulation of innate immunity and in type I IFN signal transduction. The CGGGG Indel polymorphism in the IRF5 gene is in the promoter region of the IRF5 transcript containing exon 1A, where the risk allele carries four copies of a repeated CGGGG unit. This type of IRF5 polymorphism has been generally associated with ADs, such as systemic lupus erythematosus, rheumatoid arthritis, inflammatory bowel diseases and multiple sclerosis [47]. It is worth noting that Miceli-Richard et al. [33] reported that the link with the CGGGG Indel IRF5 polymorphism was independent of the autoantibody profile of secretion.

### Negative stressful life events

According to our results, experiencing a greater number of negative stressful life events significantly increases the risk of pSS onset. In fact, it is acknowledged that many patients with pSS suffer from anxiety or depression, which are considered complications of pSS [48]. However, these negative emotions, which are commonly associated with long-term stressful life events, might also act as a key trigger for the incidence of pSS, as our results suggested. Although negative stressful life events are more likely to lead to more psychological problems, it could not be dismissed that individuals might incur physical diseases if they experience a greater number of negative stressful events or long-term stress. It is noted that a previous meta-analysis suggested that stressors may play an important role in the etiopathogenesis of ADs [49] and negative stressful events certainly constitute an important stressor. Stress mainly involves the endocrine and nervous systems, both of which are closely linked to the immune system. Skopouli et al. provided evidence in their study of the impact of stress on salivary gland epithelial cells, demonstrating that stress could become immunogenic through its diverse impact on salivary gland epithelium [50]. In clinical practice, experts generally agree that long-term stress could lead to, or aggravate, a patient’s condition. The onset of pSS is slow and insidious and may be an outcome of the accumulation of stress. Our findings highlighted the significance of a multidimensional clinical approach, which is consistent with the ethos that we should pay attention to both the psychological and physical condition of a patient. This may provide some novel insight into the pathogenesis of ADs.

### Other potential risk factors

We qualitatively analyzed and summarized 10 studies that could not be included in the quantitative synthesis because of the lack of other similar studies, and identified some other potential risk factors, including genetic risk factors, for pSS.

McCoy et al. performed the largest study to date to evaluate sex hormone exposure with pSS, and their findings suggested that female sex hormones might be protective for pSS (16), or conversely, that reduced female sex hormones might be associated with pSS. This would be consistent with the epidemiology of pSS, in that onset typically occurs during perimenopause when estrogen and progesterone levels drop [51]. Lu et al. found a remarkable increased risk of pSS in female patients with irregular menstrual cycles, especially those in their mid-forties to mid-fifties [18].

Chao et al. investigated the correlation between the use of antibiotics for non-tuberculous mycobacterial infection and the risk of SS through a population-based dataset and found that the use of new macrolides, fluoroquinolones and tetracyclines was positively associated with the development of SS, whereas the use of amikacin appeared to play a protective role [17]. This correlation was also consistent with an analysis that included individuals with a history of non-tuberculous mycobacterial infection [31]. Hence, whether the usage of antibiotics is an independent risk factor for the onset of pSS requires further evaluation.

Machowicz et al. examined the association between a Mediterranean diet and SS and found that a Mediterranean diet was linked with a lower likelihood of pSS [15]. Given the putative anti-inflammatory potential and immune protective effect of a Mediterranean diet, it might be reasonable that adherence to a Mediterranean diet, which is considered accessible, affordable and sustainable, could be a protective strategy for the general population against immune disease [52].

It is commonly agreed that pSS is an autoimmune disease with a complex genetic background. Our current study confirmed this view and, based on the five studies mentioned below, identified more than five genetic risk factors for pSS. Du et al. identified functional leucocyte immunoglobulin like receptor A3 as a susceptibility factor for pSS [19]. This factor highly predisposes to a higher risk of leucopenia and autoantibody-positive sub-phenotypes in pSS. Liu et al. studied the contribution of dendritic cell immunoreceptor polymorphisms in susceptibility to systemic lupus erythematosus and pSS and concluded that single nucleotide polymorphism (SNP) rs2377422 in dendritic cell immunoreceptor was a genetic risk factor for pSS [20]. Sun et al. reported that two SNPs (rs2736340, rs13277113) in the FAM167A-BLK region were risk factors for the development of pSS in the Han Chinese population [21]. Johannes et al. confirmed that, like other systemic ADs, FCGR3B CN was a genetic susceptibility factor for pSS [22]. Gestermann et al. provided evidence that the mRNA levels of STAT4a and type 1 IFN-induced genes were genetic risk factors for pSS, which supported the possible direct involvement of STAT4 in not only the production of type 2 IFN but also in mediating the effects of type 1 IFN [23].

### Limitations of the study

There were some limitations of our study. First, a considerable quantity of literature could not be included in this meta-analysis because of ineligible statistical methods, and this limited the supportive evidence for our findings. Second, some included studies were heterogeneous. For example, regarding infection, different studies included cases with different types of infection, and regarding smoking, the duration and frequency of exposure varied for each smoker. However, sensitivity analysis was conducted, and the results of the fixed-effects and random effects models were compared. In addition, as most of the included literature was a case–control study, the extent to which our findings could be demonstrated was limited.

## Conclusion

Our research indicated that infection, a family history of AD in FDR, a history of pregnancy, the CGGGG indel polymorphism in the IRF5 gene and negative stressful life events might be risk factors for pSS. In contrast, our study demonstrated that a history of smoking was not associated with pSS, while current smoking was negatively associated with pSS. These differences may potentially reflect early pathological changes, highlighting the chronic, insidious but progressive nature of pSS. The literature on pSS is currently limited, and more prospective, genetic and epidemiological studies on larger and more geographically diverse populations are needed to clarify the range of risk factors for pSS, which may allow for more timely and accurate patient stratification.

## Supplementary information

Below is the link to the electronic supplementary material.

Supplementary file1 (DOCX 32 KB)

Supplementary file2 (DOCX 32 KB)

## Data Availability

The original contributions presented in the study are included in the article/Supplementary Material. Further inquiries can be directed to the corresponding author.

## Footnotes

1 https://clinicaltrials.gov/
