# Supplementary material for: Risk factors for primary Sjögren’s Syndrome: a systematic review and meta-analysis
Source: Clin Rheumatol. 2022 Dec 19;42(2):327–38. doi: 10.1007/s10067-022-06474-8 (PMC9873717; doi:10.1007/s10067-022-06474-8)
Supplement: Supplementary file 2 — Supplementary file2 (DOCX 32 KB) [file 10067_2022_6474_MOESM2_ESM.docx]

**Supplementary Table 1.** Limitations and Quality assessment of NOS of excluded reports

| Author, year of publication | Limitations | Quality assessment of NOS | | | |
| --- | --- | --- | --- | --- | --- |
|  |  | Selection | Comparability | Exposure/Outcome | Total score |
| McCoy Sara S *et al*.2022[1] | 1 | *** | ** | ** | ******* |
| *Gonca et al*.2011[2] | 1 | *** | ** | ** | ******* |
| Jingxiu Xuan *et al*.2020[3] | 1 | *** | ** | * | ****** |
| Serena Colafrancesco *et al*.2019[4] | 1 | **** | * | ** | ******* |
| C. Ciccacci *et al*.2019[5] | 1,4 | ** | - | ** | **** |
| Erika Fabiola López-Villalobos *et al*.2019[6] | 1 | ** | ** | ** | ****** |
| Chien-Yu Lin et al.2019[7] | 1 | *** | ** | *** | ******* |
| Donald U. Stone *et al*.2017[8] | 4 | * | ** | * | **** |
| Zheng Junfeng *et al*.2015[9] | 2,4 | *** | - | - | *** |
| Benjamin Chaigne *et al*.2015[10] | 1 | **** | ** | ** | ******** |
| Gunnel Nordmark *et al*.2013[11] | 1 | *** | ** | * | ****** |
| Fei Sun *et al*.2013[12] | 1 | **** | ** | * | ******* |
| Yongzhe Li *et al*.2013[13] | 4 | *** | - | - | *** |
| G Nordmark *et al*. 2011[14] | 1 | *** | ** | * | ****** |
| SL Musone *et al*. 2011[15] | 1 | *** | - | ** | ***** |
| G. Kabalak *et al*.2009[16] | 1,4 | *** | - | * | **** |
| Corinne Miceli-Richard *et al*.2007[17] | 4 | *** | - | * | **** |
| Mariann Harangi *et al*.2005[18] | 3 | *** | * | *** | ******* |
| Yu-Huei Huang *et al*.2019[19] | 1 | **** | - | *** | ******* |

Note. 1, ineligible statistical method; 2, overlapping data; 3, no enough data; 4, NOS score < 5 stars

References

1. McCoy SS, Hetzel S, VanWormer JJ, Bartels CM (2022) Sex hormones, body mass index, and related comorbidities associated with developing Sjögren's disease: a nested case-control study. Clin Rheumatol 41:3065-74. doi: 10.1007/s10067-022-06226-8.
2. Karabulut G, Kitapcioglu G, Inal V, Kalfa M, Yargucu F, Keser G, et al (2011) Cigarette smoking in primary Sjögren's syndrome: positive association only with ANA positivity. Mod Rheumatol 21:602-7. doi: 10.1007/s10165-011-0446-3.
3. Xuan J, Ji Z, Wang B, Zeng X, Chen R, He Y, et al (2022) Serological Evidence for the Association Between Epstein-Barr Virus Infection and Sjögren's Syndrome. Front Immunol 11:590444. doi: 10.3389/fimmu.2020.590444.
4. Colafrancesco S, Ciccacci C, Priori R, Latini A, Picarelli G, Arienzo F, et al (2019) STAT4, TRAF3IP2, IL10, and HCP5 Polymorphisms in Sjögren's Syndrome: Association with Disease Susceptibility and Clinical Aspects. J Immunol Res 2019:7682827. doi: 10.1155/2019/7682827.
5. Ciccacci C, Latini A, Perricone C, Conigliaro P, Colafrancesco S, Ceccarelli F, et al (2019) TNFAIP3 Gene Polymorphisms in Three Common Autoimmune Diseases: Systemic Lupus Erythematosus, Rheumatoid Arthritis, and Primary Sjogren Syndrome-Association with Disease Susceptibility and Clinical Phenotypes in Italian Patients. J Immunol Res 2019:6728694. doi: 10.1155/2019/6728694.
6. López-Villalobos EF, Carrillo-Ballesteros FJ, Muñoz-Valle JF, Palafox-Sánchez CA, Valle Y, Orozco-Barocio G, et al (2019) Association of CD28 and CTLA4 haplotypes with susceptibility to primary Sjögren's syndrome in Mexican population. J Clin Lab Anal 33: e22620. doi: 10.1002/jcla.22620.
7. Lin CY, Tseng CF, Liu JM, Chuang HC, Lei WT, Liu LY, et al (2019) Association between Periodontal Disease and Subsequent Sjögren's Syndrome: A Nationwide Population-Based Cohort Study. Int J Environ Res Public Health 16:771. doi: 10.3390/ijerph16050771.
8. Stone DU, Fife D, Brown M, Earley KE, Radfar L, Kaufman CE, et al (2017) Effect of Tobacco Smoking on The Clinical, Histopathological, and Serological Manifestations of Sjögren's Syndrome. PLoS One 12: e0170249. doi: 10.1371/journal.pone.0170249.
9. Zheng J, Huang R, Huang Q, Deng F, Chen Y, Yin J, et al (2015) The GTF2I rs117026326 polymorphism is associated with anti-SSA-positive primary Sjögren's syndrome. Rheumatology (Oxford) 54:562-4. doi: 10.1093/rheumatology/keu466.
10. Chaigne B, Lasfargues G, Marie I, Hüttenberger B, Lavigne C, Marchand-Adam S, et al. (2015) Primary Sjögren's syndrome and occupational risk factors: A case-control study. J Autoimmun 60:80-5. doi: 10.1016/j.jaut.2015.04.004.
11. Nordmark G, Wang C, Vasaitis L, Eriksson P, Theander E, Kvarnström M, et al (2013) Association of genes in the NF-κB pathway with antibody-positive primary Sjögren's syndrome. Scand J Immunol 78:447-54. doi: 10.1111/sji.12101.
12. Sun F, Xu J, Wu Z, Li P, Chen H, Su J, et al (2013) Polymorphisms in the FAM167A-BLK, but not BANK1, are associated with primary Sjögren's syndrome in a Han Chinese population. Clin Exp Rheumatol 31:704-10.
13. Li Y, Zhang K, Chen H, Sun F, Xu J, Wu Z, et al (2013) A genome-wide association study in Han Chinese identifies a susceptibility locus for primary Sjögren's syndrome at 7q11.23. Nat Genet 45:1361-5. doi: 10.1038/ng.2779.
14. Nordmark G, Kristjansdottir G, Theander E, Appel S, Eriksson P, et al (2011) Association of EBF1, FAM167A(C8orf13)-BLK and TNFSF4 gene variants with primary Sjögren's syndrome. Genes Immun. 12:100-9. doi: 10.1038/gene.2010.44.
15. Musone SL, Taylor KE, Nititham J, Chu C, Poon A, Liao W, et al (2011) Sequencing of TNFAIP3 and association of variants with multiple autoimmune diseases. Genes Immun 12:176-82. doi: 10.1038/gene.2010.64.
16. Kabalak G, Dobberstein SB, Matthias T, Reuter S, The YH, Dörner T, et al (2009) Association of immunoglobulin-like transcript 6 deficiency with Sjögren's syndrome. Arthritis Rheum 60:2923-5. doi: 10.1002/art.24804.
17. Miceli-Richard C, Comets E, Loiseau P, Puechal X, Hachulla E, Mariette X (2007) Association of an IRF5 gene functional polymorphism with Sjögren's syndrome. Arthritis Rheum 56:3989-94. doi: 10.1002/art.23142.
18. Harangi M, Kaminski WE, Fleck M, Orsó E, Zeher M, Kiss E, et al (2005) Homozygosity for the 168His variant of the minor histocompatibility antigen HA-1 is associated with reduced risk of primary Sjögren's syndrome. Eur J Immunol 35:305-17. doi: 10.1002/eji.200425406.
19. Huang YH, Kuo CF, Huang LH, Hsieh MY (2019) Familial Aggregation of Psoriasis and Co-Aggregation of Autoimmune Diseases in Affected Families. J Clin Med 8:115. doi: 10.3390/jcm8010115.

**Supplementary Table 2.** Quality assessment of NOS

| Author, year of publication | Selection | Comparability | Exposure/Outcome | Total score |
| --- | --- | --- | --- | --- |
| A. Machowicz *et al*.2020[15] | *** | ** | ** | ******* |
| McCoy Sara S *et al*.2020[16] | ** | * | *** | ****** |
| Wen-ChengChao *et al*.2018[17] | **** | ** | ** | ******** |
| Ming-Chi Lu *et al*.2016[18] | **** | ** | ** | ******** |
| Yan Du *et al*.2015[19] | *** | * | ** | ****** |
| Mengru Liu *et al*.2015[20] | *** | - | ** | ***** |
| Fei Sun *et al*.2013[21] | **** | ** | * | ******* |
| JOHANNES C. NOSSENT et al.2012[22] | *** | * | * | ***** |
| N Gestermann *et al*.2010[23] | *** | - | ** | ***** |
| Behrouz Mostafavi *et al*.2005[24] | **** | ** | ** | ******** |
| FanYan Meng *et al*.2021[25] | *** | ** | *** | ******** |
| Johannes mofors *et al*.2020[26] | *** | ** | ** | ******* |
| Hadas Ben-Eli *et al*.2019[27] | *** | * | ** | ****** |
| Luisa Servioli *et al*.2019[28] | *** | ** | ** | ******* |
| J.mofors *et al*.2019[29] | ** | ** | *** | ******* |
| Peter Olsson *et al*.2017[30] | **** | ** | - | ****** |
| Wen-Cheng Chao *et al*.2017[31] | **** | ** | **** | ******** |
| Chih-Ching Yeh *et al*. 2016[32] | **** | ** | ** | ******** |
| Corinne MR *et al*. 2009[33] | *** | - | *** | ****** |
| G Nordmark *et al*.2009[34] | *** | - | *** | ****** |
| D Karaiskos *et al*.2009[35] | **** | ** | ** | ******** |
| R Priori *et al*.2007[36] | *** | * | ** | ****** |
